# Protein S blocks the extrinsic apoptotic cascade in tissue plasminogen activator/N-methyl D-aspartate-treated neurons via Tyro3-Akt-FKHRL1 signaling pathway

**DOI:** 10.1186/1750-1326-6-13

**Published:** 2011-02-03

**Authors:** Huang Guo, Theresa M Barrett, Zhihui Zhong, José A Fernández, John H Griffin, Robert S Freeman, Berislav V Zlokovic

**Affiliations:** 1 Center for Neurodegenerative and Vascular Brain Disorders, Department of Neurosurgery and Neurology, University of Rochester Medical Center, Rochester, NY 14642, USA; 2 Department of Molecular and Experimental Medicine, The Scripps Research Institute, La Jolla, CA 92037, USA; 3 Department of Pharmacology and Physiology, University of Rochester Medical Center, Rochester, NY 14642, USA

## Abstract

**Background:**

Thrombolytic therapy with tissue plasminogen activator (tPA) benefits patients with acute ischemic stroke. However, tPA increases the risk for intracerebral bleeding and enhances post-ischemic neuronal injury if administered 3-4 hours after stroke. Therefore, combination therapies with tPA and neuroprotective agents have been considered to increase tPA's therapeutic window and reduce toxicity. The anticoagulant factor protein S (PS) protects neurons from hypoxic/ischemic injury. PS also inhibits N-methyl-D-aspartate (NMDA) excitotoxicity by phosphorylating Bad and Mdm2 which blocks the downstream steps in the intrinsic apoptotic cascade. To test whether PS can protect neurons from tPA toxicity we studied its effects on tPA/NMDA combined injury which in contrast to NMDA alone kills neurons by activating the extrinsic apoptotic pathway. Neither Bad nor Mdm2 which are PS's targets and control the intrinsic apoptotic pathway can influence the extrinsic cascade. Thus, based on published data one cannot predict whether PS can protect neurons from tPA/NMDA injury by blocking the extrinsic pathway. Neurons express all three TAM (Tyro3, Axl, Mer) receptors that can potentially interact with PS. Therefore, we studied whether PS can activate TAM receptors during a tPA/NMDA insult.

**Results:**

We show that PS protects neurons from tPA/NMDA-induced apoptosis by suppressing Fas-ligand (FasL) production and FasL-dependent caspase-8 activation within the extrinsic apoptotic pathway. By transducing neurons with adenoviral vectors expressing the kinase-deficient Akt mutant *Akt^K179A ^*and a triple FKHRL1 Akt phosphorylation site mutant (FKHRL1-TM), we show that Akt activation and Akt-mediated phosphorylation of FKHRL1, a member of the Forkhead family of transcription factors, are critical for FasL down-regulation and caspase-8 inhibition. Using cultured neurons from Tyro3, Axl and Mer mutants, we show that Tyro3, but not Axl and Mer, mediates phosphorylation of FHKRL1 that is required for PS-mediated neuronal protection after tPA/NMDA-induced injury.

**Conclusions:**

PS blocks the extrinsic apoptotic cascade through a novel mechanism mediated by Tyro3-dependent FKHRL1 phosphorylation which inhibits FasL-dependent caspase-8 activation and can control tPA-induced neurotoxicity associated with pathologic activation of NMDA receptors. The present findings should encourage future studies in animal stroke models to determine whether PS can increase the therapeutic window of tPA by reducing its post-ischemic neuronal toxicity.

## Background

Thrombolytic therapy for acute ischemic stroke with a recombinant tissue plasminogen activator (tPA) has clear benefits if administered within a relatively narrow therapeutic window [1-3]. However, studies using animal models of stroke have indicated that tPA may exert serious side effects in the ischemic brain which include blood-brain barrier (BBB) breakdown [4-6] frequently resulting in intracerebral bleeding if systemic tPA is administered 3-4 h after stroke [7,8]. Moreover, a recent study using a model of angiographically documented recanalization of the rabbit middle cerebral artery occlusion has indicated that tPA produces bleeding at all doses in proportion to its thrombolytic potential [9]. These side effects limit use of tPA therapy in humans with stroke [10,11].

Studies using a transient ischemia stroke models have demonstrated direct post-ischemic neuronal toxicity of tPA [12-14]. It has been also shown that tPA enhances neuronal injury in the presence of N-methyl-D-aspartate (NMDA) [15-18]. The exact mechanism(s) how tPA interacts with the NMDA receptors (NMDARs) has, however, been debated. Never-the-less most investigators agree that pathologic activation of NMDARs contributes to neuronal death after acute excitotoxic trauma such as brain ischemia [19,20]. It has been reported that tPA enhances the neurotoxic effects of NMDA downstream to NMDARs by shifting the NMDA-induced neuronal injury from the intrinsic to the extrinsic apoptotic pathway [14]. Therefore, potential combination therapies with tPA and neuroprotective agents hold potential to increase the therapeutic window of tPA and reduce its toxic effects in brain associated with pathologic activation of NMDARs.

Protein S (PS) is a vitamin K-dependent anticoagulant plasma glycoprotein with multiple biologic functions [21]. Independent of its anticoagulant activity, PS exerts direct cellular effects [22-24]. In the central nervous system, PS is neuroprotective after a transient brain ischemia and also protects cultured neurons from hypoxia/glucose deprivation followed by reoxygenation [25]. PS enhances the BBB integrity after an ischemic insult as shown in a model of human BBB endothelial monolayers *in vitro *and after a transient ischemia in mice *in vivo *by acting on the TAM (Tyro3, Axl, Mer) receptor tyrosine kinase Tyro3 [26].

Recently, we have demonstrated that PS protects neurons from NMDA-induced excitotoxic injury by phosphorylating Bad and Mdm2 which in turn blocks the downstream steps in the intrinsic apoptotic cascade [27]. To test whether PS can protect neurons from tPA toxicity we employed a model of tPA and NMDA combined injury [14]. In contrast to NMDA alone, a simultaneous exposure of neurons to tPA and NMDA kills neurons by activating the extrinsic apoptotic pathway [14]. Bad [28,29] and Mdm2 [30,31] which control the intrinsic apoptotic cascade and p53/Bax proapoptotic pathway, respectively, are both targets of PS [27], but neither can influence the extrinsic cascade. Thus, based on the published work one cannot predict whether PS will protect neurons from tPA/NMDA injury by blocking the extrinsic pathway. Neurons express all three TAM receptors, i.e., Tyro3, Axl and Mer [32] Tyro3 and Mer were both shown to interact with PS on different cells types during physiologic and/or pathologic conditions [33-35]. Moreover, triple TAM mutants develop neuronal apoptosis [36] and PS null mice develop neuronal necrosis [37,38]. All these studies suggest that interactions between different TAM receptors and PS might be important for neuronal survival. Therefore, we also studied whether PS can activate TAM receptors during a tPA/NMDA insult.

## Results

### PS protects mouse cortical neurons from tPA/NMDA-mediated injury

**Figure 1:**
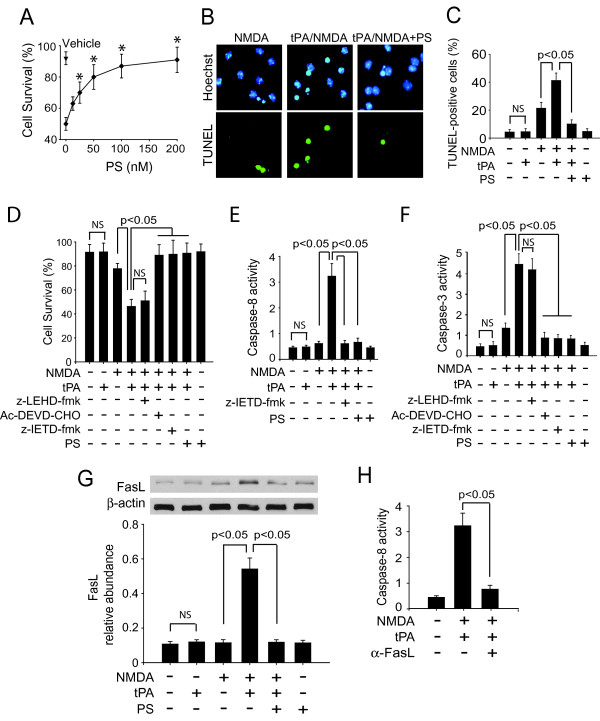
**PS protects mouse cortical neurons from tPA/NMDA-induced injury**. (A) Dose-dependent neuroprotective effects of PS (12.5-200 nM) in a 14-day old neuronal cultures 24 h after tPA/NMDA treatment. Cell survival was quantified with a WST assay. (B) Representative fluorescent-TUNEL (green)/Hoechst (blue) staining in mouse cortical neurons 24 h after tPA/NMDA treatment in the presence or absence of PS. (C) The number of apoptotic TUNEL-positive cells quantified as described in the Methods in the presence of NMDA with and without tPA or PS. (D) Survival of mouse cortical neurons 24 h after a combined tPA/NMDA treatment with and without PS, caspase-9 inhibitor (z-LEHD-fmk 5 μM), caspase-8 inhibitor (z-IETD-fmk, 50 μM), or caspase-3 inhibitor (Ac-DEVD-CHO, 50 μM). (E) Caspase-8 activity in mouse cortical neurons 8 h after tPA/NMDA exposure in the presence or absence of PS and a caspase-8 inhibitor (z-IETD-fmk; 50 μM). (F) Caspase-3 activity in mouse cortical neurons 12 h after tPA/NMDA in the presence or absence of PS, caspase-9 inhibitor (z-LEHD-fmk 5 μM), caspase-8 inhibitor (z-IETD-fmk, 50 μM), or caspase-3 inhibitor (Ac-DEVD-CHO, 50 μM). (G) Western blots for FasL in whole-cell extracts from mouse cortical neurons 6 h after tPA/NMDA in the presence or absence of PS. Intensity of FasL signal was measured by scanning densitometry and normalized to β-actin. (H) Caspase-8 activity in mouse cortical neurons 8 h after tPA/NMDA in the presence of anti-FasL antibody (α-FasL; 10 μg/ml). Anti-FasL antibody was added simultaneously with tPA-NMDA. Caspase inhibitors were applied 1 h prior to tPA/NMDA treatment. In all studies murine PS was used at 100 nM, unless specified differently, and was added simultaneously with tPA (20 μg/ml) and NMDA (25 μM). Mean ± SEM, n = 3 independent cultures in triplicate. *p < 0.05 compared with values in the absence of PS. NS, non-significant.

Mouse recombinant PS dose-dependently enhanced cell survival (Figure 1A) and reduced by > 75% the number of TUNEL-positive cells (Figure 1B-C) in 14-day old cultures of mouse cortical neurons treated with tPA and NMDA, as described in the Methods and previously reported [14,15]. Because tPA/NMDA exposure has been shown to trigger caspase-8-dependent cell death [14], we explored whether PS attenuates tPA/NMDA-induced toxicity by blocking the extrinsic apoptotic pathway. Initial experiments showed that treatment with caspase-8 and caspase-3 inhibitors (z-IETD-fmk, Ac-DEVD-CHO), but not caspase-9 inhibitor (z-LEHD-fmk), enhanced cell survival (Figure 1D), confirming that tPA/NMDA-induced neuronal cell death is caspase-8-dependent and that caspase-8 is upstream to caspase-3, as reported [14]. Consistent with these results, activation of both caspase-8 and caspase-3 in tPA/NMDA-treated neurons was suppressed by the caspase-8 inhibitor z-IETD-fmk, but not by a caspase-9 inhibitor (Figure 1E-F). Treatment with PS (100 nM) blocked the tPA/NMDA-mediated activation of both caspase-8 and caspase-3. In these studies PS was added at concentration of 100 nM because the maximal protection with murine PS in the present model has been achieved with 100 nM (Figure 1A).

In the extrinsic cell death pathway, activation of caspase-8 is coupled to activation of death receptors by proteins such as FasL [39]. Indeed, tPA/NMDA treatment increased FasL expression (Figure 1G). Moreover, addition of an anti-FasL neutralizing antibody at the time of tPA/NMDA exposure prevented activation of caspase-8 (Figure 1H). tPA/NMDA exposure failed to elicit an increase in FasL expression in cells treated with PS (Figure 1G) suggesting that this may be the mechanism by which PS prevents caspase-8 activation. Treatment with tPA alone and PS alone did not affect the number of TUNEL-positive cells, cell survival, caspase-8 and caspase-3 activities, and FasL production (Figure 1C-G). In this model, treatment with NMDA alone caused less severe neuronal injury compared to tPA/NMDA combination (Figure 1C-D) and did not affect caspase-8 activity (Figure 1E), consistent with a previous report [14].

### PS neuroprotection requires activation of Akt signaling

**Figure 2:**
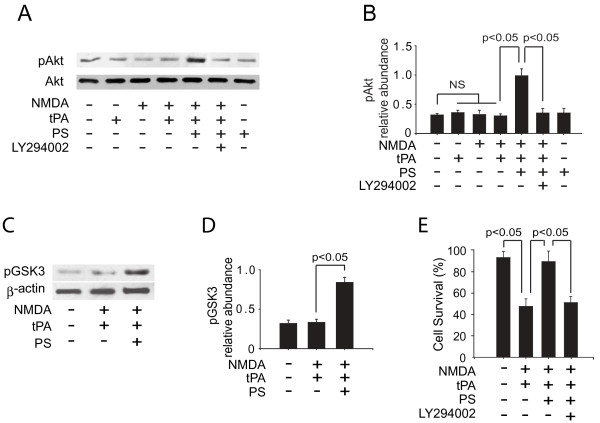
**PS protects tPA/NMDA-treated neurons via activation of PI3K/Akt signaling**. **(**A) Western blots for pAkt (Ser473) and total Akt in whole-cell extracts from mouse cortical neurons 2 h after tPA/NMDA exposure in the presence or absence of PS and LY294002 (50 μM). (B) Intensity of pAkt signal was measured by scanning densitometry and normalized to total Akt. (C) Akt activity was determined 2 h after tPA/NMDA exposure in the presence or absence of PS using an immune-complex kinase assay with GSK3 as the substrate. (D) Intensity of the pGSK3 signal was normalized to β-actin. (E) Survival of mouse cortical neurons 24 h after tPA/NMDA exposure in the presence or absence of PS and LY294002 (50 μM). LY294002 was added to the culture 1 h before tPA/NMDA treatment. In all studies, murine PS was used at 100 nM and was added simultaneously with tPA/NMDA. Mean ± SEM, n = 3 independent cultures in triplicate. NS, non-significant.

PS requires activation of the phosphatidylinositol 3-kinase (PI3K)/Akt survival pathway as an upstream signaling event to prevent NMDA-mediated neuronal injury [27]. Thus, we tested whether the ability of PS to protect neurons from tPA/NMDA-mediated injury also requires PI3K/Akt activation as an initial step mediating the observed neuronal protection. PS treatment stimulated phosphorylation of Akt on Ser473 [40] in neurons after tPA/NMDA challenge (Figure 2A-B). Because phosphorylation levels of Akt may not entirely reflect its protein kinase activity, we performed an immune complex kinase assay using glycogen synthase kinase 3 (GSK3) as the Akt substrate [41]. Results from these experiments confirmed that PS treatment stimulates Akt kinase activity (Figure 2C-D). LY294002, a PI3K inhibitor, blocked PS-mediated phosphorylation of Akt (Figure 2A-B) and neuronal protection (Figure 2E) after tPA/NMDA exposure. This data suggests that PS's neuroprotective effects requires an initial activation of the PI3K/Akt pathway.

**Figure 3:**
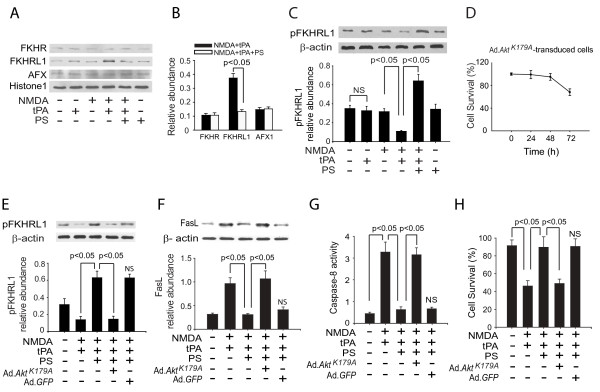
**PS-mediated neuroprotection requires Akt phosphorylation of FKHRL1**. (A) Western blots for FKHR, FKHRL1 and AFX in nuclear fractions from mouse cortical neurons treated with tPA/NMDA for 4 h in the presence or absence of PS. (B) Signals for FKHR, FKHRL1 and AFX were normalized to histone H1. (C) Western blots for pFKHRL1 in whole-cell extracts from mouse cortical neurons prepared 4 h after tPA/NMDA treatment in the presence or absence of PS. Intensity of pFKHRL1 signal was measured by scanning densitometry and normalized to β-actin. (D) Time-dependent survival (12-72 h) of neurons after transduction with Ad. *Akt^K179A^*. Cell survival was quantified with a WST assay. (E) Western blots for pFKHRL1 in whole-cell extracts prepared from neurons transduced with Ad.*Akt^K179A ^*or Ad.*GFP *and exposed for 4 h to tPA/NMDA in the presence or absence of PS. Intensity of the pFKHRL1 signal was normalized to β-actin. (F) Western blots for FasL in whole-cell extracts from neurons transduced with Ad.*Akt^K179A ^*or Ad.*GFP *and treated for 6 h with tPA/NMDA in the presence or absence of PS. Intensity of the FasL signal was normalized to β-actin. (G) Caspase-8 activity in neurons transduced with Ad.*Akt^K179A ^*or Ad.*GFP *and exposed to tPA/NMDA for 8 h in the presence or absence of PS. (H) Cell survival of neurons transduced with Ad.*Akt^K179A ^*or control Ad.*GFP *and exposed to tPA/NMDA for 24 h in the presence or absence of PS. In all studies murine PS was used at 100 nM and was added simultaneously with tPA/NMDA. Mean ± SEM, n = 3 independent cultures in triplicate. NS, non-significant.

Activated Akt promotes cell survival in the extrinsic apoptotic pathway by phosphorylating and inactivating FKHRL1, a member of the Forkhead family of transcription factors [42]. Phosphorylation of FKHRL1 results in its cytoplasmic retention, whereas dephosphorylated FKHRL1 translocates into the nucleus where it stimulates transcription of its target genes including FasL in the extrinsic apoptotic pathway [40,42]. Thus, we determined the protein levels of three members of the Forkhead family, FKHRL1, FKHR and AFX [43-45] in nuclear fractions prepared from tPA/NMDA-treated neurons. Nuclear levels of FKHRL1, but not of FKHR and AFX1, were significantly increased by tPA/NMDA challenge (Figure 3A-B). PS treatment blocked the increase in nuclear FKHRL1 (Figure 3A-B), and this was accompanied by an increase in the amount of FKHRL1 phosphorylated on Ser253 (a site phosphorylated by Akt [42]) in whole cell lysates from tPA/NMDA-treated neurons (Figure 3C). However, PS did not affect the levels of nuclear FKHRL1 and phosphorylated FKHRL1 in neurons without tPA/NMDA challenge (Figure 3A, C).

To confirm whether Akt plays a key role in PS-mediated neuronal protection, mouse cortical neurons were transduced with a recombinant adenovirus expressing a kinase-deficient Akt mutant (Ad.*Akt^K179A ^*) [46] along with GFP. The transduction efficiency was ~70% as determined by GFP fluorescence (data not shown). Figure 3D shows that mouse cortical neurons transduced with Ad.*Akt^K179A ^*did not affect cell survival within 48 h of transduction. Ad.*Akt^K179A ^*expression, but not control Ad.*GFP*, abolished PS-stimulated phosphorylation of FKHRL1 (Figure 3E), down-regulation of FasL (Figure 3F), inhibition of caspase-8 (Figure 3G) and neuronal protection (Figure 3H) in neurons exposed to tPA/NMDA.

**Figure 4:**
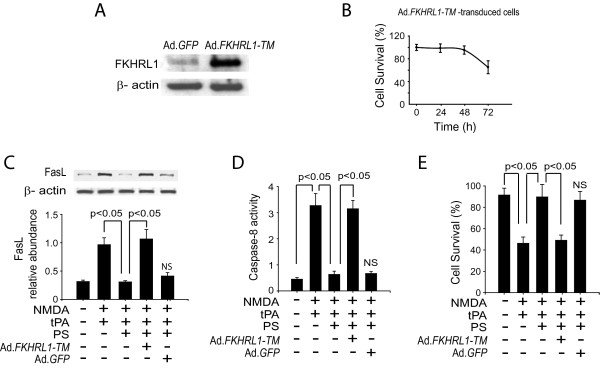
**PS-mediated Akt phosphorylation of FKHRL1 suppresses FasL production and activation of caspase-8**. (A) Expression of FKHRL1 and FKHRL1-TM (triple mutant) in mouse cortical neurons 24 h after adenoviral transduction determined by total FKHRL1 immunoblotting analysis. (B) Time-dependent survival (12-72 h) of neurons after transduction with Ad.*FKHRL1-TM*. Cell survival was quantified with a WST assay. (C) Western blots for FasL in whole-cell extracts from neurons transduced with Ad.*FKHRL1-TM *or control Ad.*GFP *and treated for 6 h with tPA/NMDA in the presence or absence of PS. Intensity of the FasL signal was normalized to β-actin. (D) Caspase-8 activity in neurons transduced with Ad.*FKHRL1-TM *or Ad.*GFP *and exposed to tPA/NMDA for 8 h in the presence or absence of PS. (E) Cell survival of neurons transduced with Ad.*FKHRL1-TM *or Ad.*GFP *and exposed to tPA/NMDA for 24 h in the presence or absence of PS. In all studies murine PS was used at 100 nM and was added simultaneously with tPA/NMDA. Mean ± SEM, n = 3 independent cultures in triplicate. NS, non-significant.

We then tested the importance of phosphorylation of FKHRL1 in PS-mediated neuronal protection. Mouse cortical neurons were transduced with recombinant adenovirus expressing a triple mutant of FKHRL1 (Ad. *FKHRL1-TM*), which cannot be phosphorylated by Akt [47]. The transduction efficiency was ~70% as assessed by GFP fluorescence (data not shown). Figure 4A illustrates the level of expression of FKHRL1-TM in mouse cortical neurons compared to endogenous FKHRL1 in control neurons. Since neurons expressing *FKHRL1-TM *may undergo apoptosis [48], we examined the viability of neurons transduced with Ad.*FKHRL1-TM*. Our data showed that mouse cortical neurons transduced with Ad.*FKHRL1-TM *did not undergo spontaneous apoptosis within 48 h of transduction, although they did show about 35% decrease in survival at 72 h (Figure 4B). Thus, in subsequent experiments we transduced neurons with Ad.*FKHRL1-TM *24 h before tPA/NMDA treatment. Results from these experiments showed that Ad.*FKHRL1-TM *expression abolishes PS-mediated down-regulation of FasL (Figure 4C), inhibition of caspase-8 (Figure 4D) and neuronal protection (Figure 4E), suggesting that FKHRL1 is a critical downstream step in the PS-mediated blockade of the extrinsic apoptotic pathway.

### Tyro3 mediates PS neuroprotection and FKHRL1 phosphorylation

**Figure 5:**
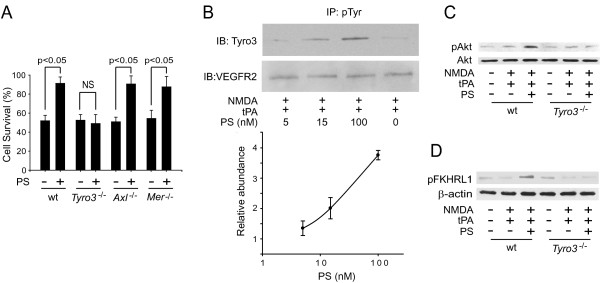
**PS neuroprotection requires Tyro3 activation**. (A) Cell survival of cortical neurons isolated from Tyro3, Axl or Mer null mice and wild type (wt) mice after 24 h of tPA/NMDA exposure in the presence or absence of PS. Cell survival was quantified with a WST-8 assay. (B) PS dose-dependently mediates Tyro3 tyrosine phosphorylation in tPA/NMDA-treated mouse cortical neurons as determined by immunoprecipitation (IP) with anti-phospho-tyrosine (pTyr) antibody followed by immunoblotting (IB) with anti-Tyro3 antibody and anti-VEGFR2 (vascular endothelial growth factor receptor 2) antibody (as a loading control) 2 h after tPA/NMDA. Graph, relative abundance of phosphorylated Tyro3 to VEGFR2. (C) Akt phosphorylation (pAkt, Ser473) in neurons isolated from Tyro3 null mice and wt controls exposed to tPA/NMDA for 2 h in the presence or absence of PS. (D) FKHRL1 phosphorylation (pFKHRL1, Ser253) in neurons isolated from Tyro3 null mice and wt controls exposed to tPA/NMDA for 4 h in the presence or absence of PS. In all studies murine PS was used at 100 nM unless specified and was added simultaneously with tPA/NMDA. Mean ± SEM, n = 3 independent cultures in triplicate. NS, non-significant.

Next, we tested the role of TAM receptors in PS protection of tPA/NMDA-treated neurons. Using cortical neurons from *Tyro3^-/-^*, *Axl^-/- ^*and *Mer^-/- ^*mice, we found that PS protects neurons lacking Axl and Mer from tPA/NMDA-mediated injury, but failed to protect neurons lacking Tyro3 (Figure 5A). Consistent with a model whereby PS acts through Tyro3, we found that mouse PS stimulated tyrosine phosphorylation of Tyro3 on mouse cortical neurons challenged by tPA and NMDA (Figure 5B). However, PS failed to activate Akt (Figure 5C) and phosphorylate FKHRL1 (Figure 5D) in tPA/NMDA-treated neurons from Tyro3 null mice.

## Discussion

It has been recently reported that PS phosphorylates the TAM receptor Tyro3 in neurons which protects NMDA-treated neurons from cell death by inhibiting the intrinsic apoptotic cascade [27]. Specifically, it has been shown that PS acts on Tyro3 which activates the PI3K/Akt pathway in NMDA-treated neurons resulting in phosphorylation of Bad and Mdm2 which in turn increases the levels of antiapoptotic Bcl-2 and Bcl-X_L _proteins and reduces the levels of proapoptotic p53 and Bax, respectively, thus inhibiting the intrinsic apoptotic cascade initiated by NMDA [27]. However, in the present model of a combined tPA/NMDA injury, a simultaneous exposure to tPA and NMDA activates the extrinsic apoptotic cascade but does not affect or amplify the intrinsic apoptotic pathway, as we reported [14]. Moreover, the established phosphorylation targets of PS in the intrinsic apoptotic cascade, i.e., Bad [28,29] and Mdm2 [30,31] as shown by a previous study [27], do not influence the extrinsic cascade.

The present study shows for the first time that PS can block the extrinsic apoptotic cascade and protect neurons from a combined tPA/NMDA-mediated injury. We also show that inhibition of the extrinsic apoptotic cascade by PS requires Tyro3-mediated phosphorylation of FKHRL1 which in turn inhibits FasL production and FasL-dependent caspase-8 activation in the extrinsic pathway. These results suggest that PS may protect neurons from divergent inducers of apoptosis acting through either intrinsic or extrinsic pathways, similar as reported for activated protein C [49]. By using Tyro3-, Axl- and Mer-deficient neurons, the present study demonstrates that an initial upstream event in PS-mediated protection against tPA/NMDA combined injury requires Tyro3 and activation of the PI3K/Akt anti-apoptotic signaling pathway as in an NMDA alone-mediated neuronal injury [27]. However, the present study in tPA/NMDA-treated neurons establishes the link between PS-mediated activation of Tyro3 and FKHRL1 phosphorylation which inactivates pro-apoptotic FKHRL1 [42] and mediates PS's beneficial effects. Namely, dephosphorylated FKHRL1 can trigger the extrinsic apoptotic cell death by promoting expression of FasL followed by FasL-dependent activation of caspase-8 [40,42]. Our data using a triple Akt-phosphorylation site mutant of FKHRL1 (i.e., FKHRL1-TM) [47] indicate that FKHRL1 is a critical target of Akt after neuronal injury involving activation of the extrinsic cascade. Moreover, our data shows that PS-mediated inactivation of FKHRL1 suppresses tPA/NMDA-induced FasL up-regulation which subsequently blocks caspase-8 activation.

The importance of the extrinsic apoptotic cascade for tPA/NMDA-mediated toxicity is further supported by observation that >80% of caspase-3 activation in tPA/NMDA-treated neurons was blocked by a caspase-8 inhibitor, but not with a caspase-9 inhibitor, similar as shown in a previous study [14]. Both pro-caspase-3 and Bid, a proapoptotic promoter of cytochrome c release [50], are substrates for caspase-8. However, it has been shown that tPA/NMDA-induced apoptotic signaling does not require amplification through the intrinsic pathway via Bid [14] in contrast to ischemic neuronal death *in vivo *[50,51]. Therefore, our results are consistent with a model in which PS blocks FasL and FasL-dependent activation of caspase-8 followed by inhibition of caspase-3 activation resulting in protection of tPA/NMDA-treated neurons.

In addition to the effects of a combined tPA/NMDA injury on signaling pathways downstream to NMDARs, as shown in the present and a previous study [14], it has been also suggested that tPA may interact directly with NMDARs. For example, it has been proposed that tPA cleaves the NR1 subunit of NMDARs potentiating NMDA-induced calcium influx and neurotoxicity [15,17,52] or that NR2D-containing NMDARs mediate tPA-induced neuronal excitotoxicity [16], but this has not been confirmed by other groups [18]. It has been reported that seizures induced by ethanol-withdrawal are controlled by tPA through modulation of NR2B-containing NMDARs [53]. Other studies have indicated that tPA binding to the low-density lipoprotein receptor related protein-1 (LRP1) is necessary for NMDARs activation [54] and upregulation of matrix metalloproteinase-9 expression at the BBB [55] resulting in an increased BBB permeability [4,56] which in turn may lead to accumulation in brain of blood-derived neurotoxins and vascular-mediated neuronal injury [57], as shown for example in pericyte-deficient mouse models [58,59]. Whether PS can act directly on NMDARs or LRP1 to attenuate the neurotoxic signaling during a tPA/NMDA-mediated insult should be addressed by the future studies.

## Conclusions

Our data suggests that PS blocks the extrinsic apoptotic cascade through Tyro3-dependent phosphorylation of FKHRL1 which in turn inhibits FasL-dependent caspase-8 activation and controls tPA-induced neuronal toxicity associated with pathologic activation of NMDA receptors. These findings should encourage future preclinical studies in different animal stroke models using different species and different administration regimens of tPA and PS to determine whether PS can increase the therapeutic window of tPA by reducing its risk for intracerebral bleeding and/or post-ischemic neuronal toxicity.

## Methods

### Reagents

Full-length properly gamma-carboxylated mouse recombinant PS was prepared and characterized as we reported [23]. NMDA was purchased from Sigma (St Louis, MO, USA). Mouse recombinant tPA was purchased from Molecular Innovations (Novi, MI, USA). For western blot analysis the following antibodies were used: polyclonal rabbit antibody against mouse Fas Ligand (FasL; EMD4Biosciences, Gibbstown, NY, USA), polyclonal rabbit antibody against mouse Akt (Cell Signaling, Danvers, MA, USA), polyclonal rabbit antibody against mouse pAkt (Cell Signaling), polyclonal rabbit antibody against human FKHR which cross reacts with mouse FKHR (Cell Signaling), polyclonal rabbit antibody against human FoxO3a which cross reacts with mouse FoxO3a (FKHRL1, Cell Signaling), polyclonal rabbit antibody against human pFoxO3a which cross reacts with mouse pFoxO3a (pFKHRL1, Cell Signaling), polyclonal rabbit antibody against human AFX which cross reacts with mouse AFX (Cell Signaling), and polyclonal sheep antibody against human histone 1, which cross reacts with mouse histone 1 (United States Biological, Swampscott, MA, USA). The inhibitory peptides, Ac-DEVD-CHO (caspase-3), z-IETD-fmk (caspase-8) and z-LEHD-fmk (caspase-9) were selected on the basis of substrate specificity [14] and purchased from Sigma. LY294002 was purchased from Cell Signaling. Anti-FasL neutralizing antibody was selected as reported [60,61] and purchased from BD Pharmingen (clone MFL3; San Diego, CA, USA).

### Transgenic mice

TAM transgenic mice (*Tyro3^-/-^*, *Axl^-/-^*, *Mer^-/-^*) were originally on C57B16/129 background [62,36]. These mice were backcrossed for 10 generations to attain the C57B16 background and were generated by null-null breeding. The C57B16 mice were used as wild type controls for the TAM null mice, as reported [[63]; please see also the Jackson Laboratory website http://jaxmice.jax.org/strain/007937.html]. The breeding pairs were originally provided by Dr. G. Lemke from the Salk Institute for Biological Studies, La Jolla, CA.

### Mouse neuronal cell cultures

Primary neuronal cultures were established as described [49]. In brief, cerebral cortex was dissected from embryonic day 16 from C57BL/6J mice and different TAM null mice, treated with trypsin for 10 min at 37°C and dissociated by trituration. Dissociated cell suspensions were plated at 3.5 × 10^5 ^cells per well on 12-well tissue culture plates or at 1.5 × 10^6 ^cells per well on 6-well tissue culture plates coated with poly-L-lysine, in serum-free Neurobasal medium plus B27 supplement (Gibco, Rockville, MA, USA). The medium suppresses glial growth to < 0.5% of the total cell population, as demonstrated by immunocytochemistry for glial fibrilary acidic protein [64,65]. Cultures were allowed to mature for 14 days *in vitro *before treatment as reported [14]. Medium was replaced every 3 days.

### tPA/NMDA injury model

For induction of neuronal apoptosis, neuronal cultures were treated with mouse recombinant tPA (20 μg/ml) and 25 μM NMDA/5 μM glycine in serum-free Neurobasal medium plus B27 supplement for 2-24 h as reported [14,15].

### Detection of apoptosis

Apoptotic cells were visualized by *in situ *terminal deoxynucleotidyl transferase-mediated digoxigenin-dUTP nick-end labeling (TUNEL) assay according to manufacturer's instructions (Promega, Madison, WI, USA). Cells were counterstained with the DNA-binding fluorescent dye, Hoechst 33342 (Molecular Probes, Eugene, OR, USA) at 1 μg/ml for 10 min at room temperature to reveal nuclear morphology. Images were obtained using a Zeiss 510 meta confocal microscope. The number of apoptotic cells was expressed as the percentage of TUNEL-positive cells of the total number of nuclei determined by Hoechst staining.

### Cell survival assay

Neuronal viability was detected by using a 2-(2-methoxy-4-nitrophenyl)-3-(4-nitrophenyl)-5-(2,4-disulfophenyl)-2H-tetrazolium monosodium salt (WST-8) assay (Dojindo Molecular Technologies, Gaithersburg, ML, USA). The cell survival rate was expressed as the viability percentage of the vehicle-treated cells.

### Caspase-9, caspase-8 and caspase-3 activities

The caspase-9, caspase-8 and caspase-3 activities in cell lysates were determined using caspase-9, caspase-8 and caspase-3 Colormetric Assay Kits (Chemicon, Rosemont, IL). Approximately 50 μg of protein was incubated with DEVD-pNA (for caspase-3; 50 μM), IETD-pNA (for caspase-8; 50 μM) or LEHD-pNA (for caspase-9; 50 μM) and 10 mM DTT at 37°C for 2 h. Substrate hydrolysis was determined as absorbance change at 405 nm in a microplate reader. Enzymatic activity was expressed in arbitrary units (optical density, OD) per mg protein.

### Western blot analysis

Whole cellular extracts and nuclear protein fractions were prepared and protein concentration was determined by using Bradford protein assay (Bio-Rad, Hercules, CA, USA); 10-50 μg of protein was analyzed by 10% SDS-PAGE and transferred to nitrocellulose membranes that were then blocked with 5% nonfat milk in TBS (100 mM, Tris pH 8.0, 1.5 M of NaCl, 0.1% Tween 20) for 1 h. The membranes were incubated overnight with primary antibodies, washed with TBS, and incubated with a horseradish peroxidase-conjugated secondary antibody for 1 h. Immunoreactivity was detected by using the ECL detection system (Thermo Scientific, Rockford, IL, USA).

### Akt kinase assay

We followed instructions in the Akt kinase assay kit from Cell Signaling Technology (catalog # 9840). Cells were washed with PBS and lysed in cell lysis buffer. Twenty microliters of immobilized Akt primary antibody bead slurry was added to 200 μl of whole-cell extract (150 μg of protein) overnight at 4°C. After centrifugation, the supernatant was saved for β-actin detection by western blot to confirm that the same amount of whole-cell extract was used for the Akt kinase assay as reported [41]. Immunoprecipitates were washed three times with lysis buffer and twice with Akt kinase buffer. Kinase assays were performed for 30 min at 30°C under continuous agitation in kinase buffer containing 200 μM ATP, 1 μg of GSK-3 fusion protein. Samples were analyzed by western blot using phospho-GSK-3α/β (Ser21/9) antibody.

### Ad.^AktK179A ^and Ad.FKHRL-TM constructs

The kinase-inactive Akt^K179A ^[46] or a triple mutant of FKHRL1 (FKHRL1-TM) [47] was cloned into a GFP-containing adenoviral vector using AdEasy ™XL system (Stratagene, Cedar Creek, TX, USA). The adenoviral product containing Akt^K179A ^or FKHRL1-TM was amplified in HEK 293A cells from American Type Culture Collection (ATCC, Manassas, VA, USA) and purified using ViraKit ™(Virapur, San Diego, CA, USA). Cortical neurons were transduced with adenoviral constructs (200 MOI) 24 h before studies. The transduction efficiency was determined by GFP signal and western blot analysis.

### Tyro3 tyrosine phosphorylation

Neurons were lysed with Radio ImmunoPreciptation Assay (RIPA) Buffer (50 mM Tris, pH 8.0, 150 mM NaCI, 0.1% SDS, 1.0% NP-40, 0.5% sodium deoxycholate and Roche protease inhibitor cocktail) and incubated with a rabbit anti-phospho-tyrosine antibody (Abcam, Cambridge, MA) or a control non-immune IgG (Sigma-Aldrich) overnight at 4°C. The samples were then immunoprecipitated using a protein G immunoprecipitation kit (Roche) followed by SDS-PAGE separation and transfer onto nitrocellulose membranes (Millipore Corp). After blocking non-specific sites with 5% milk, the membranes were incubated with a rat monoclonal anti-mouse Tyro3 antibody (R&D Systems) or a rabbit anti-mouse vascular endothelial growth factor receptor 2 (VEGFR2) antibody (Millipore, Billerica, MA) for a loading control. Following incubation with an HRP-conjugated donkey-anti goat secondary antibody (Santa Cruz Biotechnology), the immunoreactivity was detected using the SuperSignal^® ^West Pico chemiluminescent substrate (Thermo Scientific). Cells were treated with mouse PS for 2 h.

### Statistical analysis

We used S-plus 7.0 for statistical calculations. Data are presented as mean ± SEM. Student's *t*-test and one-way analysis of variance (ANOVA) followed by Tukey's post-hot test were used to determine statistically significant differences. *P *< 0.05 was considered statistically significant.

## Abbreviations

PS: Protein S; NMDA: N-methyl-D-aspartate; NMDAR: N-methyl-D-aspartate receptor; TAM: Tyro3, Axl, Mer; tPA: tissue plasminogen activator; FasL: Fas-ligand; BBB: blood-brain barrier; PI3K: phosphatidylinositol 3-kinase; GSK3: glycogen synthase kinase 3.

## Competing interests

The authors declare that they have no competing interests.

## Authors' contributions

HG supervised most of the experiments, performed data analysis and helped with the manuscript preparation. TMB carried out most of the experiments. ZZ performed the Tyro3 tyrosine phosphorylation assay and carried out studies with the adenoviral contstructs. JAF provided and characterized mouse recombinant PS. JHG contributed to study design working with BVZ. RSF provided *Ad.^AktK179A ^*and *Ad.FKHRL-TM *constructs and provided critical comments to the manuscript. BVZ designed the entire study, supervised all portions of the study, and wrote the manuscript. All authors read and approved the final manuscript.
